# Synergistic interaction between lead and cadmium via metal-transporter gene upregulation in mice

**DOI:** 10.1007/s10534-026-00829-3

**Published:** 2026-05-17

**Authors:** Tingting Zhang, Weiqin Xing, James A. Ippolito, Shuangshuang Shao, Xinjun Huang, Yongqiang Yang, Linlin Zhao, Yongxia Cheng, Liping Li

**Affiliations:** 1 School of the Environment, Henan University of Technology, Zhengzhou, 450001 Henan China; 2 School of Environment and Natural Resources, The Ohio State University, Columbus, OH 43210 USA; 3 Henan International Joint Laboratory of Environmental Pollution, Remediation and Food Quality Security, Zhengzhou, 450001 Henan China; 4 School of Environmental and Bioengineering, Henan University of Engineering, Zhengzhou, 451191 Henan China; 5 Jiyuan Ecological and Environmental Monitoring Center of Henan Province, Jiyuan, 459000 Henan China

**Keywords:** Lead, Cadmium, Synergism, Transporter, Gene, Expression

## Abstract

This study investigated the potential synergistic interactions between Pb and Cd, and assessed the contribution of transporter gene expression to the synergism. Mice were administered varying doses of Pb (15, 30 and 108 mg kg^−1^) or Cd (1, 2 and 3 mg kg^−1^), or a combination of both in the feed. Liver, kidney and bone samples were analyzed for Cd, Pb, Cu, Mn, Zn and Fe concentrations. For treatments exhibiting synergistic effects, the relative expression levels of the divalent metal transporter 1 (*Dmt1*), ZRT-IRE-like protein 8 (*Zip8*) and *Zip14* were quantified in the liver and kidney using Quantitative Real-Time Reverse Transcription Polymerase Chain Reaction (qRT-PCR). At 108 mg kg^−1^ Pb**,** increasing Cd doses from 0 to 1 mg kg^−1^ significantly increased Pb concentrations in the kidney, liver and bone by 10.6, 60.2 and 19.6%, respectively; concurrently, the relative expression of the *Zip8* in the kidney increased by 69.5%, while *Dmt1* and *Zip14* expression in the liver increased by 79.0 and 85.6%, respectively. At Cd doses ≥ 1 mg kg^−1^, increasing Pb rate resulted in elevated Cd concentrations in the kidney, liver and bone; however, no significant changes in transporter expression levels were observed in the kidney under these treatments, possibly due to the upregulation of transporter genes in the intestines. Results demonstrate that interactions between Pb and Cd can be synergistic, and upregulation of certain transporter genes were responsible for such synergism. These findings offer important insights for mitigating the health risks associated with heavy metal exposure in animal and humans.

**Supplementary Information:**

The online version contains supplementary material available at 10.1007/s10534-026-00829-3.

## Introduction

Human exposure to heavy metals usually involves co-exposure to multiple metals, such as Cd, Cu, Pb, Zn and Hg, as accumulation of these metals are often found in soils, dusts and food (Shim et al. 2017; Li et al. 2022). Heavy metal ions of similar charge and size in animals can interact with each other during absorption, transport, and binding with proteins. Interactions between heavy metals in humans can be antagonistic (Li et al. 2023a, 2019; Bulat et al. 2008) or synergistic (Ollson et al. 2017; Li et al. 2023a, 2023b; Bulat et al. 2017). The antagonistic effect of one heavy metal on another can inhibit the absorption of the latter, or both (Li et al. 2019).

However, synergistic effects have also been observed between metals, including Pb and Cd (Ollson et al. 2017; Li et al. 2019, 2023a, 2023b). Adding soluble Cd and Pb to mouse feed amended with a mining/smelting metal-contaminated soil resulted in significantly higher kidney Pb concentrations at certain doses compared with feed spiked with metal-contaminated soil alone (Li et al. 2019). Ollson et al. (2017) found that, compared with mice exposed to Pb alone in the feed, co-exposure to Cd and Pb significantly increased liver Pb concentrations. Our previous study also found that chickens fed diets spiked with Cd and Pb contaminated soils had greater organ Pb or Cd concentrations than mice fed diets spiked with soluble Pb or Cd at the similar levels (Li et al. 2023a), possibly due to synergistic interactions between metals in the soil.

Interactions between cationic metals can also occur on the transporter synthesis level, with elevated concentration of one metal potentially upregulating or downregulating the expression of a transporter gene which can transport both metals (Ohta and Ohba 2020; Gebeyew et al. 2022), thus resulting in altered concentrations of the second metal within organisms. For example, under Zn- and Fe-sufficient conditions, the body maintains homeostasis by downregulating the expression of the Zn transporter ZIP (Zrt-/Irt-like protein) family proteins (such as ZIP8 and ZIP14) and the iron transporter DMT1, all of which possess a strong affinity for Cd and Mn (Gunshin et al. 1997; Jenkitkasemwong et al. 2012; Yanatori and Kishi 2019). This subsequently decreases the number of entry pathways for Cd into cells, leading to reduced Cd accumulation. In addition, heavy metal accumulation can also be affected by the altered synthesis of binding proteins under exposure of another metal. It has been found that Zn-induced synthesis of metallothioneins (MTs) promoted the sequestration of Cd within the cytoplasm, which effectively restricted Cd diffusion and mitigated Cd toxicity (Zhang et al. 2019; Ryu et al. 2004; Park et al. 2001).

Mechanism(s) of synergism between heavy metals in animals may be due to the compensatory upregulation of metal transporters (Ollson et al. 2017). Under co-exposure to Cd and Pb, Cd competes with iron (Fe) for the divalent metal transporter 1 (DMT1), thereby reducing cellular Fe uptake. To maintain cellular Fe homeostasis, DMT1 expression is upregulated, which in turn increases Pb accumulation via the DMT1 pathway (Garrick et al. 2006; Ollson et al. 2017). The enhanced DMT1 expression is partly due to DMT1 having a stronger affinity for Cd than for Fe (Garrick et al. 2006; Ollson et al. 2017). At present, synergistic interactions among heavy metals in animals have been observed in only a few studies. Therefore, it is necessary to confirm the existence of such synergisms and to investigate whether the mechanism underlying the potential synergism between Pb and Cd is associated with the expression of transporter genes*.* Thus, in this mouse bioassay study, we examined alterations in the relative expression levels of metal transporters associated with the synergistic interactions occurring in mouse organs.

## Materials and methods

### Mice and mice feed

The experiments for this study were conducted at School of the Environment, Henan University of Technology (Zhengzhou, Henan, China). Female Kunming (KM) mice (*Mus musculus*) (5 weeks old and specified pathogen-free) were used in the experiment. Both the mice and commercial feed were bought from Liaoning Changsheng Biotechnology Co., Ltd (Shenyang, Liaoning, China). According to the manufacturer’s specifications, the concentrations of Cu, Zn, Mn, and Fe in the diet were approximately 10, 30, 75 and 120 mg kg^−1^, respectively. The mouse feeding experiment was designed and conducted according to the guide for the Care and Use of Laboratory Animals (National Research Council 2011), and received approval from the Research Ethics Committee of Henan University of Technology (as per customary practice, no formal approval number was issued). No anesthetics were used in this experiment.

The feed was oven-dried at 60 °C and then ground to powder (< 0.05 mm) using a food shredder (Philips HR1704, Zhuhai, Guangdong, China). The experiment was comprised of sixteen feeding treatments of either Pb, Cd, or a combination of Pb and Cd (Table S1). To increase the likelihood of synergistic effect occurrence between Pb and Cd in mice, the metal doses used in this study were selected based on mouse heavy metal exposure levels in previous studies that reported Pb and Cd synergisms (Olsson et al., 2017; Li et al. 2023a). The powdered feed was mixed with metal solutions (Pb(Ac)_2_ and CdCl_2_ for Pb and Cd, respectively), moistened with deionized water gradually, mixed thoroughly, then made into rods (2 cm diameter by 5 cm length) manually. The feeds of all treatments were then freeze-dried. A portion each treatment was oven-dried, digested using EPA 3051A method (US EPA, 2007), and then the digestate heavy metal concentrations were determined using Inductively Coupled Plasma Mass Spectrometry (ICP-MS) (8900 G3665A, Agilent, CA, USA). The feed heavy metal concentrations are presented in Table S1.

### Mice feeding and sample collection

A total of 192 mice were used in the experiment. The mice were randomly assigned to 16 groups (corresponding to 16 treatments (Table S1)), each consisting of 4 cages, with 3 mice per cage. The mice were raised with 12/12 light/dark cycles at 20–22 °C, and relative humidity between 50 and 60%. Initially, the mice underwent a 7-day acclimatization period, during which they were provided with ad libitum access to the commercial feed and deionized water. Following acclimatization, the feed and water were removed, and the mice were fasted overnight. The body weight (BW) of the mice was recorded using the cage as the unit of observation and analysis. The mice were then fed the prepared feeds (Table S1) for 10 days with ad libitum access to feed and deionized water. Feed and water consumption of each cage was recorded each morning. At the conclusion of the 10-day exposure, the mice were fasted overnight, weighed, and then euthanized using the cervical dislocation method. The entire liver, both kidneys, and bone (mixture of femur and tibia) samples were collected from each mouse. A portion of the mouse organ/tissue samples were stored at − 40 ℃, while the remaining portion was immediately stored at − 80 ℃ for mRNA expression analysis after collection.

### Sample heavy metal concentration analysis

The samples stored at − 40 °C were freeze-dried (FD-1A-50, BiLon, Shanghai, China), and muscles were removed from the bone after freeze-drying. The kidney, liver and bone samples were then ground and homogenized, digested using EPA 3051A method (US EPA, 2007), and the digestate Cd, Cu, Mn, Ni, Pb, Fe and Zn concentrations were then determined using ICP-MS.

### RNA extraction and cDNA synthesis

After heavy metal concentration analysis, kidney and liver samples with synergistic effects were chosen for RNA extraction; however, the T9 and T12 pair was excluded from measurement because of an unintentional omission. Total RNA from the − 80 °C mouse kidney and liver samples was extracted using the Trizol reagent (Avramov et al. 2024). The RNA concentration and purity were measured using a micro-nucleic acid analyzer (Nanodrop-2000, Thermo Fisher Scientific, Waltham, MA, US). A portion of the RNA was assessed for integrity via agarose gel electrophoresis. All primers were synthesized by Shanghai Yansu Technology Co., Ltd (Shanghai, China). The reverse transcription reaction mixture was prepared using 1 µg of total RNA, 1 µL of StarScript III All-in-one RT Mix, 4 µL of 5 × StarScript III All-in-one RT Buffer and diethyl pyrocarbonate (DEPC)-treated water to a final volume of 20 µL. After thorough mixing of the reverse transcription reaction mixture, the samples were briefly centrifuged. Following centrifugation, the reaction conditions were as follows: 37 °C for 2 min, 50 °C for 15 min and 85 °C for 2 min. The resulting cDNA was either used immediately for subsequent experiments or stored frozen.

### Gene expression profile

Divalent metal transporter 1 (DMT1), ZIP8 and ZIP14 are involved in Cd and Mn transport (Fujishiro et al. 2012; Yanatori and Kishi 2019). The genes expression of these three transporters were measured, the *β-actin* (ACTB) gene was used as a stable reference gene in the experimental treatments and for normalizing the data. Primer sequences and amplicon information for the target genes are presented in Table S2. The expression of the above genes was analyzed by qRT-PCR using a Roche LightCycler480 quantitative PCR machine (Rotkreuz, Switzerland), a Biometra GmbH PCR (Model 844–070-882) instrument (Jena, Germany) and a 2 × M5 Hi Per SYBR Premix Es Taq (Tli RNaseH Plus) detection kit (MF787-01, Beijing Polymeric, Beijing, China). The two-step amplification program was as follows: initial denaturation at 95 ℃ for 2 min; each cycle consisted of 5 s denaturation at 95 °C followed by 30–34 s annealing/extension at 57 °C, for a total of 45 cycles. Three replicate samples were prepared for each treatment and three technical replicates were prepared for each sample. The mRNA expression level of the control group was set to 1, and the differences in gene expression between the experimental and control groups were analyzed.

### Quality control and quality assurance

All plastic and glassware used were soaked in a 5% HNO_3_ acid bath for > 4 h and washed completely with deionized water before use. Quality control samples analyzed with each digestion batch included reagent blanks and wheat flour (GBW(E)100, 493) and pork liver (GBW10051(GSB-29)) for the feed and mouse organ/tissue samples, respectively. The recoveries of Pb and Cd in the reference materials were 90.2–105% and 95–115%, respectively. All of the samples were analyzed in triplicate.

### Data analyses

Values of the triplicates of each sample were averaged to obtain the mean of each sample, and the mean values of the four replicates were used to calculate the mean values of each treatment. Data was analyzed using ANOVA followed by Tukey’s post hoc test in SPSS version 22.0 (SPSS Inc., Chicago, IL, USA), with data variability expressed as the standard error of the mean. A *p* < 0.05 was considered statistically significant.

## Results

### Organ/tissue heavy metal concentrations

**Fig. 1:**
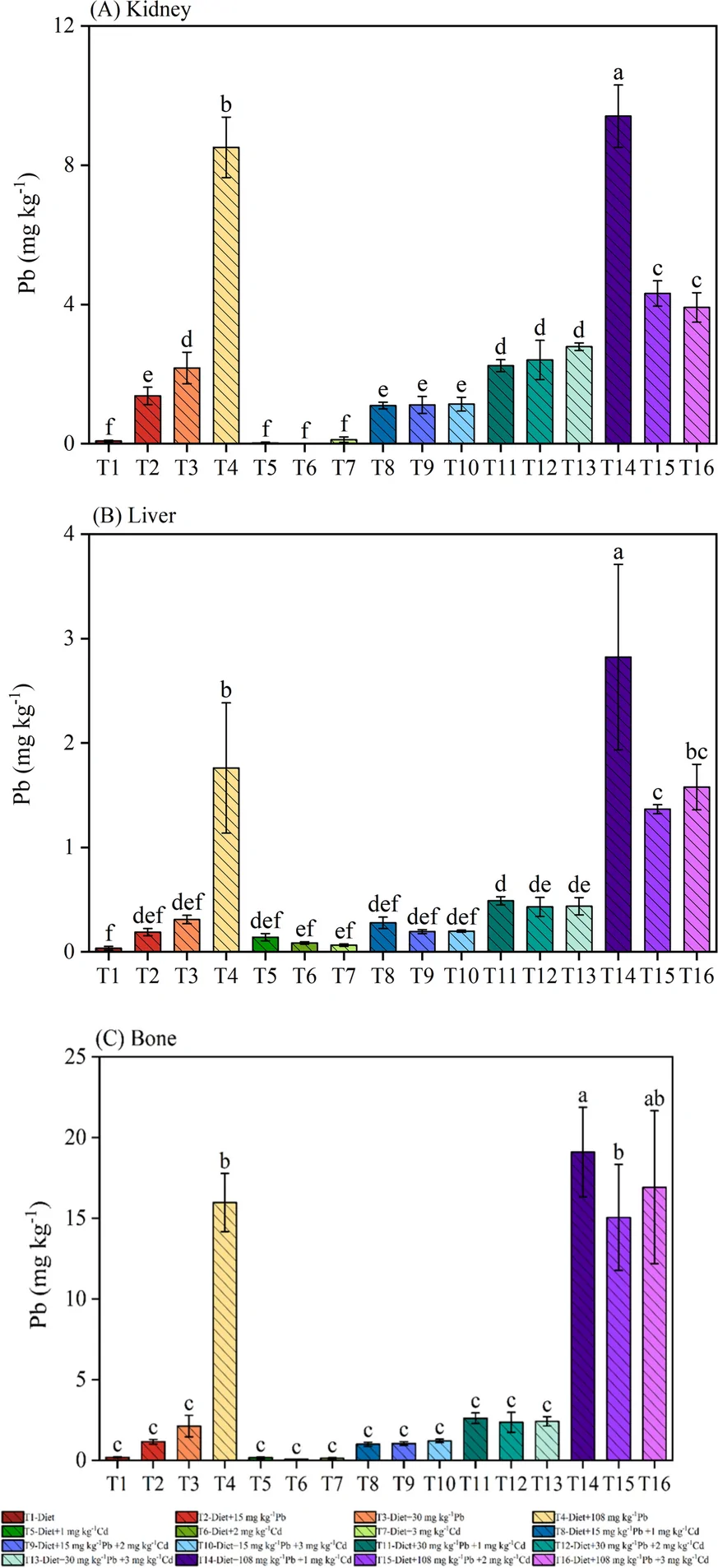
Kidney (**A**), liver (**B**), and bone (**C**) Pb concentrations of mice fed with feed spiked with different levels of Pb. Bars represent the means while error bars represent the standard error of the mean. Different letters of the same metal in the same organ/tissue indicate significant difference at *p* < 0.05

Mouse organ/tissue Pb concentrations of different treatments are presented in Fig. 1. The feed Pb/Cd spiking significantly affected mouse organ/tissue Pb concentrations. Greater Pb spiking rates resulted in greater kidney (Fig. 1a), liver (Fig. 1b) and bone (Fig. 1c) Pb concentrations. Maximum Pb concentrations at each Cd dose were all found in treatments with the greatest Pb dose (108 mg kg^−1^) for all three organ/tissues. The maximum Pb concentrations ranged from 0.00–9.41, 0.0353–2.82 and 0.0577–19.1 mg kg^−1^ for kidney, liver and bone, respectively.

For the same Pb spiking rate, different Cd rates only resulted in significant differences at the Pb rate of 108 mg kg^−1^ for all three organ/tissues (treatments T4, T14, T15 and T16). At 108 mg kg^−1^ Pb level, the kidney Pb concentrations of the 2 (T15) and 3 (T16) mg kg^−1^ Cd spiking treatments were 49.4 and 53.9% lower than the 0 mg kg^−1^ Cd (T4) treatment, and 54.2 and 58.4% lower than the 1 mg kg⁻^1^ Cd (T14) treatment (*p* < 0.05), respectively; the liver Pb concentrations of treatment T15 (108 mg kg^−1^ Pb + 2 mg kg^−1^ Cd) were 22.4% lower than treatment T4 (108 mg kg^−1^ Pb), and the liver Pb concentrations of treatments T15 and T16 (108 mg kg⁻^1^ Pb + 3 mg kg^−1^ Cd) were 51.6% and 44.1% lower than treatment T14 (108 mg kg⁻^1^ Pb + 1 mg kg⁻^1^ Cd), respectively; at the 108 mg kg⁻^1^ Pb level, the Cd rates of 2 (T15) and 3 mg kg⁻^1^ (T16) resulted in significantly lower kidney Pb concentrations than the 0 mg kg⁻1 Cd treatment (T4) and the 1 mg kg⁻^1^ Cd treatment (T14). Likewise, the Cd rate of 2 mg kg⁻^1^ (T15) resulted in a significantly lower liver Pb concentration than the 0 mg kg⁻^1^ Cd treatment (T4), and the Cd rates of 2 (T15) and 3 mg kg⁻^1^ (T16) resulted in significantly lower liver Pb concentrations than the 1 mg kg⁻^1^ Cd treatment (T14); in the bone, no significant difference was found between treatments T15 or T16 and T4 for Pb concentrations. Compared with the 0 mg kg^−1^ Cd and 108 mg kg^−1^ Pb treatment (T4), the kidney, liver, and bone Pb concentrations of the 1 mg kg^−1^ Cd + 108 mg kg^−1^ Pb treatment (T14) were 9.56, 60.3 and 19.6% greater, respectively.

**Fig. 2:**
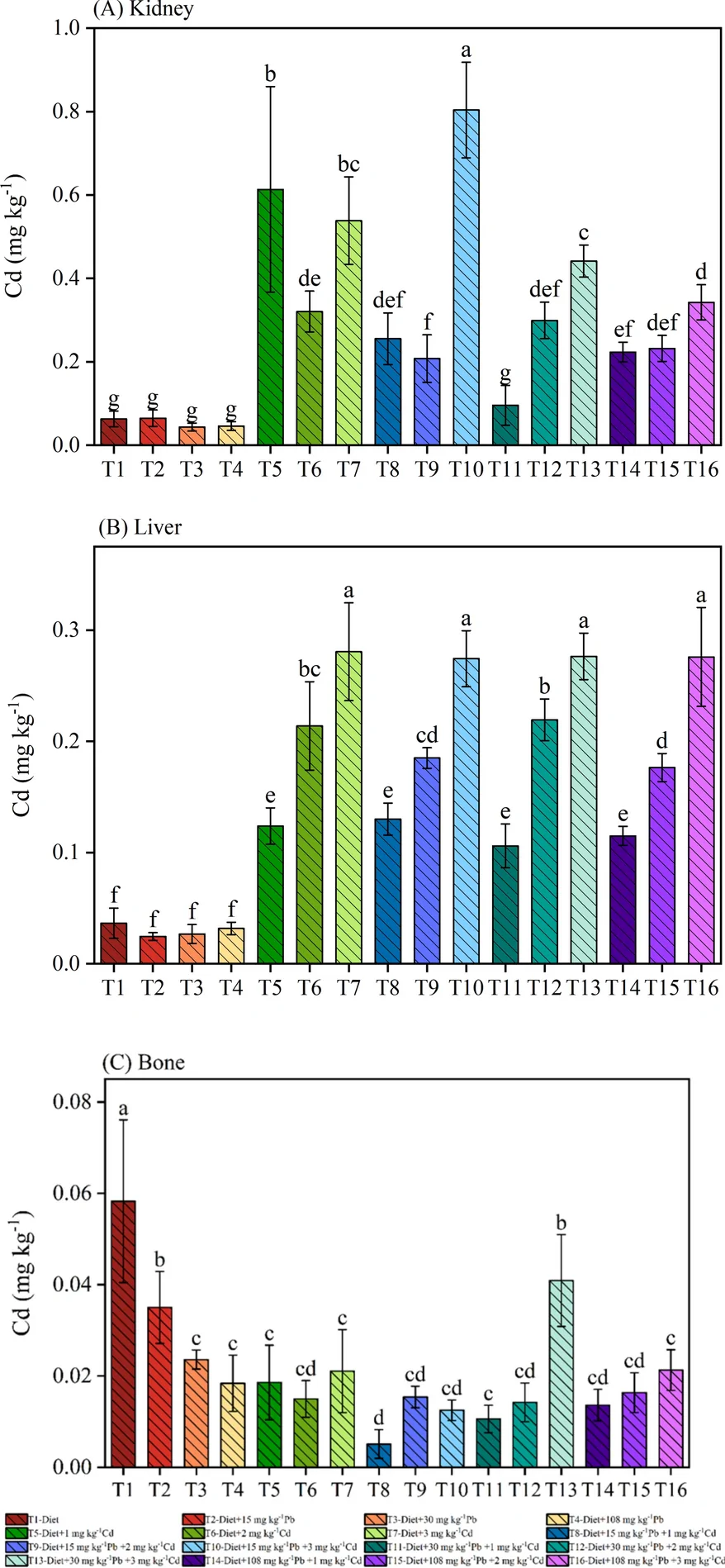
Kidney (**A**), liver (**B**) and bone (**C**) Cd concentrations of mice fed with feed spiked with different levels of Cd. Bars represent the means while error bars represent the standard error of the mean. Different letters of the same metal in the same organ/tissue indicate significant difference at *p* < 0.05

Mouse organ/tissue Cd concentrations of different treatments are presented in Fig. 2. Compared with the no-Cd treatment, Cd spiking resulted in increased Cd concentrations in the kidney (Fig. 2a) and liver (Fig. 2b). For the Cd-spiked treatments, maximum Cd concentrations at each Pb dose were all found in treatments with the greatest Cd dose (3 mg kg^−1^) for kidney and liver. The maximum Cd concentrations ranged from 0.044–0.804, 0.0318–0.280 and 0.0051–0.0583 mg kg^−1^ for kidney, liver and bone, respectively.

Compared with the liver and bone, feed Pb/Cd spiking had a more pronounced effect on Cd concentrations in the kidney (Fig. 2A). In most cases, for treatments with the same Cd doses, treatments with greater Pb doses had lower kidney Cd concentrations. However, at 1 mg kg^−1^ Cd (treatments T8, T11 and T14), when Pb doses rose from 30 (T11) to 108 mg kg^−1^ (T14), the kidney Cd concentrations increased by 132% (0.096 versus 0.223 mg kg^−1^) (*p* < 0.05); at 3 mg kg^−1^ Cd dose (treatments T7, T10 and T13), co-administration with 15 mg kg^−1^ Pb (T10) significantly increased kidney Cd levels by 49.2% compared with the treatment without Pb (T7) (0.539 versus 0.804 mg kg^−1^).

For the liver, at the same Pb doses, treatments with greater Cd doses often had greater liver Cd concentrations, while Pb doses did not affect Cd concentrations in the liver except at 2 mg kg^−1^ Cd (T6, T9, T12 and T15); liver Cd concentration of T12 (2 mg kg^−1^ Cd + 30 mg kg^−1^ Pb) was 18.4% greater than T9 (2 mg kg^−1^ Cd + 15 mg kg^−1^ Pb). For the bone, at the same Pb doses, Cd doses did not result in significant changes in Cd concentrations except for in treatments T11-T13. In most cases, no significant difference was found for Cd concentrations between treatments with different Pb doses while the same Cd doses, or treatments with greater Pb doses had lower bone Cd concentrations. However, at 3 mg kg^−1^ Cd, the treatment with 30 mg kg^−1^ Pb (T13) had significantly greater bone Cd concentrations than treatments T7, T10 and T16; bone Cd concentrations of T13 were 70.1, 187 and 68.5% greater than those of T7, T10 and T16, respectively.

The mouse kidney, liver and bone Cu, Zn, Mn and Fe concentrations are presented in Table S3. With the Cd and Pb spiking in the feed, the concentrations of Cu, Zn and Mn in the mouse organs decreased compared with T1, with some differences statistically significant. However, kidney Fe concentrations increased with Pb/Cd spiking; kidney concentrations of treatment T5 (1 mg kg^−1^ Cd) and T7 (3 mg kg^−1^ Cd) were significantly greater than that of T1. Similarly, the liver Fe concentrations of T7, T8, T13, T14 T15 and T16 were significantly greater than that of T1.

Organ/tissue heavy metal concentration correlation results are presented in Fig. S1. Significant positive correlations were found between Cu and Mn, Zn and Mn, Cu and Zn, and Cd and Fe in both kidney and liver, while in the bone, positive correlations were found between Cu and Mn, Fe and Mn, and Fe and Zn. Significant nonlinear correlations were found in all three organ/tissues between the Fe/Cd ratio and the organ/tissue Cd concentrations (Fig. S2).

### Lead dose response with different endpoints

Dose–response curves of Pb concentration with the endpoints of kidney, liver, bone, and liver + kidney + bone in mice are presented in Fig. S3. For the kidney (Fig. S3A), significant linear correlations between kidney Pb concentration and Pb dose levels at all four Cd doses were found, and the equation coefficients decreased with increasing Cd dose. The same type of response between liver or bone Pb concentration and Pb dose level at all four Cd doses were also observed (Fig. S1B ,C).

### Cadmium dose response with different endpoints

Dose–response curves of Cd concentration with endpoints of kidney, liver and bone in mice are presented in Fig. S4. For the kidney (Fig. S4A), at 15, 30 and 108 mg kg^−1^ Pb, significant linear correlations between the kidney Cd concentrations and Cd dose levels were found and the equation coefficients decreased with increasing Pb dose. For the liver (Fig. S4B), significant linear correlations were found between the liver Cd concentrations and Cd dose levels at all four Pb doses, yet the equation coefficients were relatively similar across Pb dose. Similar to the kidney, significant linear correlations between bone Cd concentration and Cd dose levels were found for Pb doses of 15, 30 and 108 mg kg^−1^ (Fig. S4C).

### Expression of metal transporter genes

**Fig. 3:**
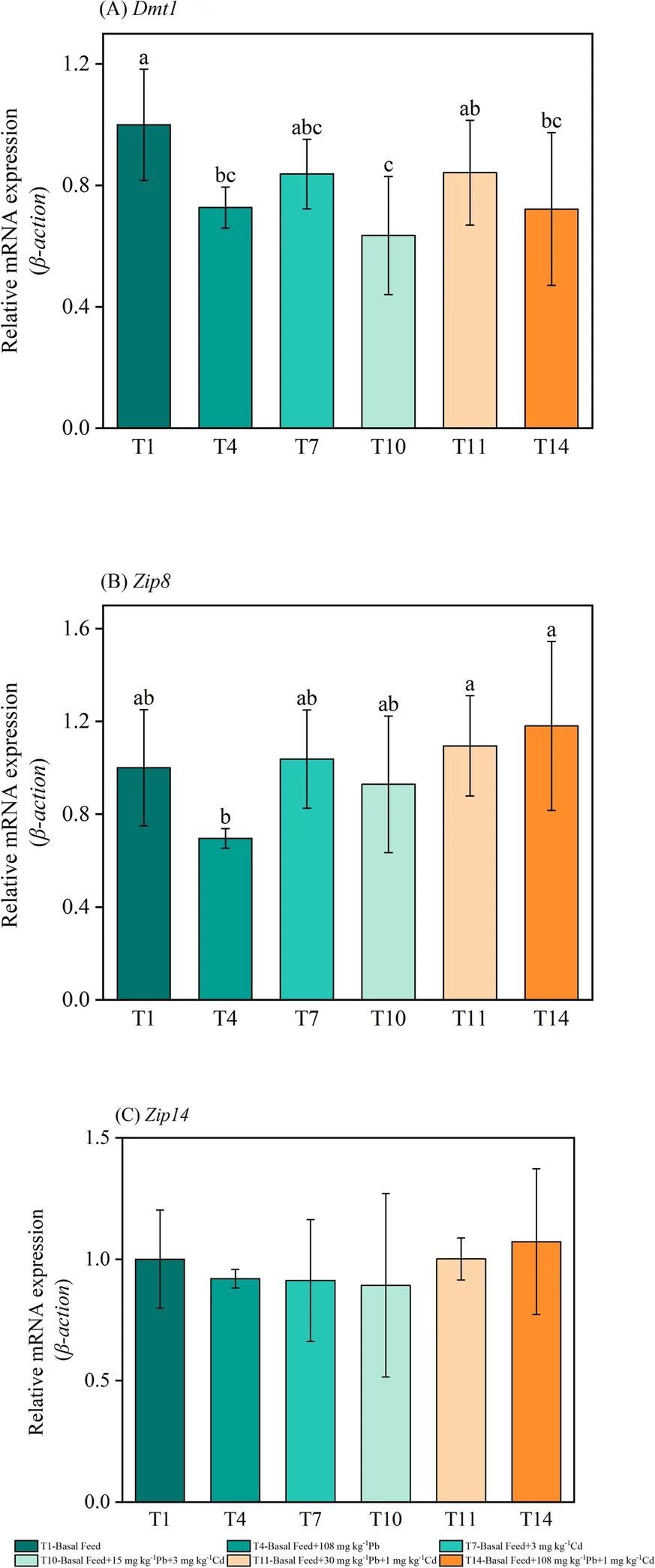
Relative expression of *Dmt1* (**A**), *Zip8* (**B**) and *Zip14* (**C**) protein genes in mouse kidney. Bars represent the means while error bars represent the standard error of the mean. Different letters of the same gene in the same organ/tissue indicate significant difference at *p* < 0.05

**Fig. 4:**
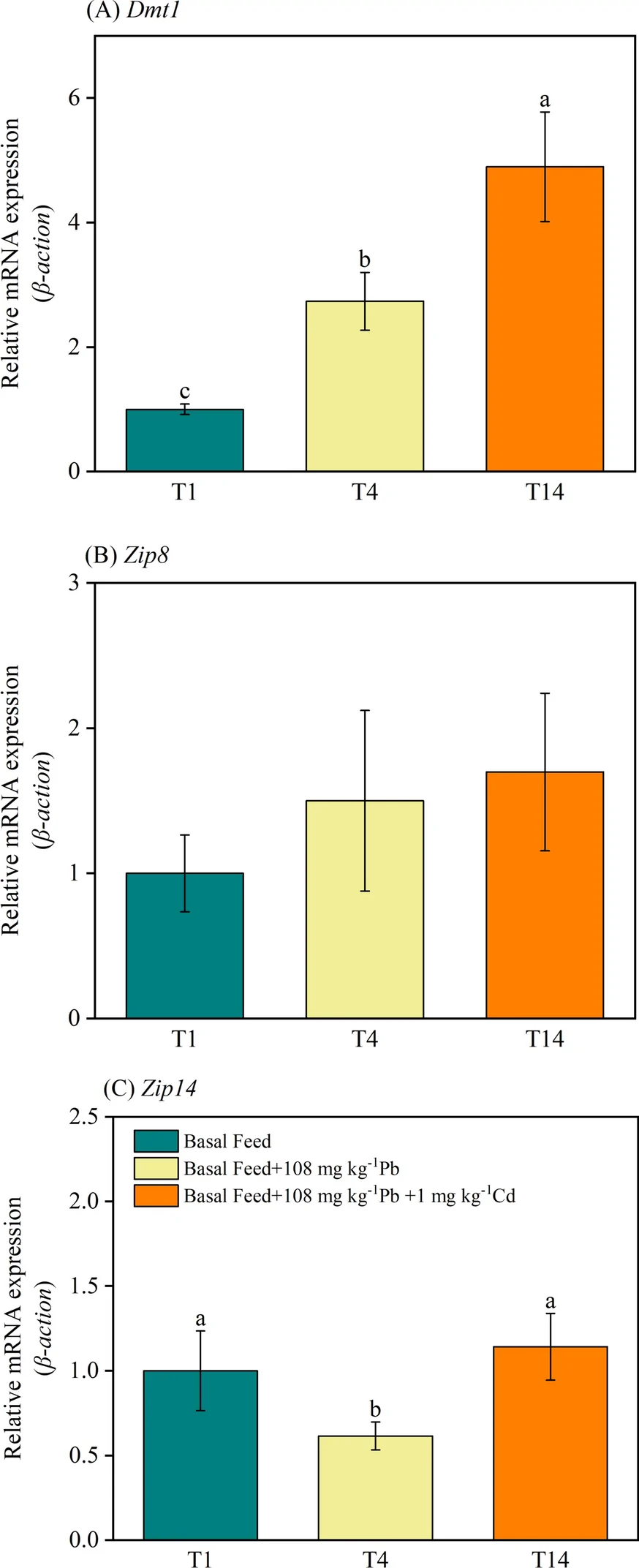
Relative expression of *Dmt1* (**A**), *Zip8* (**B**) and *Zip14* (**C**) protein genes in mouse liver. Bars represent the means while error bars represent the standard error of the mean. Different letters of the same gene in the same organ/tissue indicate significant difference at *p* < 0.05

Eight pairs of treatments exhibited Pb and Cd synergistic effects (Table 1). The synergistic effect increased Pb or Cd concentration by between 10.6 and 227%. The qRT-PCR gene relative expression assay results in the kidney and liver of these treatments are presented in Fig. 3 and Fig. 4. Compared with the control (T1), the kidney *Dmt1* gene expressions of Pb/Cd spiked treatments were significantly lower or was not upregulated (Fig. 3A), and no significant difference was found between treatment pairs with synergistic effects (Group I, II, III, Table 1). For the *Zip8* gene expression, compared with the 108 mg kg^−1^ Pb alone treatment (T4), expressions were significantly greater in the 108 mg kg^−1^ Pb + 1 mg kg^−1^Cd treatment (T14, 69.5%) (Group I in Table 1), and 30 mg kg^−1^ Pb + 1 mg kg^−1^ Cd treatment (T11, 57.3%); however, no significant difference was found between other treatment pairs (Group II, III) with synergistic effects for kidney *Zip8* gene expressions (Table 1). The expression of *Zip14* in the kidney (Fig. 3C) did not differ significantly between the treatment pairs in which synergisms were observed in Table 1.

**Table 1:** Treatments of Pb and Cd and their concentrations in mice kidney, liver, and bone when synergistic effects were observed

Organ	Group	Treatment	Added metal spiked	Organ Cd	Organ Pb	Synergized metal, % increase
			mg kg^−1^			
Kidney	I	T4	Pb 108, Cd 0	0.0463 ± 0.0052	8.51 ± 0.55b	Pb, 10.6%
		T14	Pb 108, Cd 1	0.223 ± 0.012	9.41 ± 0.45a	
Kidney	II	T7	Pb 0, Cd 3	0.539 ± 0.043b	0.116 ± 0.031	Cd, 49.2%
		T10	Pb 15, Cd 3	0.804 ± 0.047a	1.14 ± 0.10	
Kidney	III	T11	Pb 30, Cd 1	0.0959 ± 0.0238b	2.24 ± 0.09	Cd, 132%
		T14	Pb 108, Cd 1	0.223 ± 0.012a	9.41 ± 0.45	
Liver	IV	T4	Pb 108, Cd 0	0.0318 ± 0.0028	1.76 ± 0.33b	Pb, 60.2%
		T14	Pb 108, Cd 1	0.115 ± 0.004	2.82 ± 0.46a	
Liver	V	T9	Pb 15, Cd 2	0.185 ± 0.009b	0.194 ± 0.018	Cd, 18.4%
		T12	Pb 30, Cd 2	0.219 ± 0.019a	0.431 ± 0.092	
Bone	VI	T4	Pb 108, Cd 0	0.0184 ± 0.0031	16.0 ± 0.9b	Pb, 19.6%
		T14	Pb 108, Cd 1	0.0136 ± 0.0017	19.1 ± 1.4a	
Bone	VII	T7	Pb 0, Cd 3	0.0211 ± 0.0046b	0.125 ± 0.021	Cd, 93.8%
		T13	Pb 30, Cd 3	0.0409 ± 0.0050a	2.41 ± 0.14	
Bone	VIII	T10	Pb 15, Cd 3	0.0125 ± 0.0011b	1.20 ± 0.05	Cd, 227%
		T13	Pb 30, Cd 3	0.0409 ± 0.0050a	2.41 ± 0.14	

Different letters in the same column for the same organ/tissue and same group indicate significant difference at *p* < 0.05. Data are presented as mean ± SE

In the liver, compared with the control (T1), the *Dmt1* gene expressions were significantly greater in the 108 mg kg^−1^ Pb alone treatment (T4, 173%) and the 108 mg kg^−1^ Pb + 1 mg kg^−1^ Cd treatment (T14, 390%); the *Dmt1* expression in treatment T14 was significantly greater than T4 by 79% (Fig. 4A) (Group IV in Table 1). The expression of *Zip8* in the liver (Fig. 4B) did not differ significantly between the treatments T1, T4 and T14. For *Zip14* expression, the value of treatment T14 was significantly greater than T4 by 85.6% (Fig. 4C) (Group IV in Table 1). Comparison of the synergistic effect between Pb and Cd in the kidney and liver and their relationships with upregulated genes are summarized in Table S4.

## Discussion

### Synergism between Pb and Cd

The interactions among cationic heavy metals are predominantly antagonistic, mainly due to the competition for shared transporters (Andjelkovic et al. 2019; Li et al. 2019; Yu et al. 2021). In this work, synergistic effects of Cd on Pb, or Pb on Cd, were observed at certain Pb and Cd doses (Table 1) as a function of Cd and Pb concentrations in mouse kidney, liver and bone (Fig. 1and Fig. 2).

In earlier studies, synergistic effects among cationic heavy metals were found for Zn or Cd on Pb in drinking water of wood mice as indicated by kidney and bone Pb concentrations (Cooke et al. 1990), for Zn on Pb in soil as indicated by Pb RBA assayed with swine (Wijayawardena et al. 2017), for Cd on Pb in mouse feed as indicated by liver Pb concentrations (Ollson et al. 2017; Li et al. 2019), and for Pb on Cd in soils fed to chicken as indicated by Cd concentrations in chicken kidney and gizzard (Li et al. 2023a). Similar to the synergistic effect of Pb on Cd found in this work, Andjelkovic et al. (2019) found that rat kidney Cd concentration was positively affected by Pb spiking in the feed.

In the present work, the mice were exposed to four levels of both Pb (0, 15, 30 and 108) and Cd (0, 1, 2 and 3 mg kg^−1^). Based on the organ/tissue heavy metal concentrations, synergistic effects were observed for both Cd on Pb, and Pb on Cd. However, the synergistic effect of Cd on Pb in liver, kidney and bone only occurred at the Pb dose of 108 mg kg^−1^ in the feed, while the synergistic effect of Pb on Cd occurred at Cd doses of 1, 2 and 3 mg kg^−1^ in the feed, predominantly at the highest dose of 3 mg kg^−1^ Cd (Table 1). Such results indicate that the synergism between metals may be affected by their dosage, and synergism tends to occur at greater doses of the synergized metal. In Ollson et al. (2017), a synergistic effect was found for Cd on Pb accumulation in mice at greater Pb doses (15 and 30 mg kg^−1^ in feed), whereas no such effect was not observed for lower Pb dose (3 mg kg^−1^), in agreement with results of the present work. Furthermore, Ollson et al. (2017) found that lower Cd doses (0.2 mg kg^−1^) had no synergistic effect on the liver Pb concentration, while greater doses (1 and 2 mg kg^−1^) did impact liver Pb concentration. A recent work by Singh et al. (2025) found that when a mixture of seven heavy metals (Cd, Cr, Fe, Hg, Mn, Ni and Pb) were administered to mice, liver Cd concentrations increased marginally when the drinking water heavy metal concentrations increased from 1 to 5X (five times of the 1X), but when the drinking water heavy metal concentrations increased from 10 to 50X, the liver Cd concentrations increased from ~ 0.22 to ~ 1.38 mg kg^−1^. These results may have implied that antagonism on Cd by other heavy metals occurs at lower metal doses, while synergism occurs at higher doses. In plants, it has been suggested that the interactions between heavy metals were concentration-specific (Chaney and Hornick 1977; Kabata-Pendias 2010), and synergism occurs at greater doses while antagonism occurs at lower doses (Sharma et al. 1999). Further research is needed to verify such a speculation in mammals.

Based on organ/tissue heavy metal concentrations in the current work, a synergistic effect of Cd on Pb was found in all three organ/tissues at the same Pb (108 mg kg^−1^) and Cd doses (0 and 1 mg kg^−1^), while a synergistic effect of Pb on Cd was found in kidney, liver and bone yet occurred at different combination scenarios of Pb and Cd doses (Table 1). These results infer that there are differences for heavy metal accumulation mechanisms between different organ/tissues in mice, as found in Yu et al. (2009), Ollson et al. (2017) and Aydemir and Cousins (2018).

The greater kidney and liver Fe concentrations of some of Cd/Pb exposed treatments, as compared to the control (Table S3), indicated that Fe accumulation in the mouse was enhanced under the Cd and Pb exposure. This occurred in Cd-only exposure in the kidney, or in the liver at > 2 mg kg^−1^ Cd doses in Cd-only exposures and at 108 mg kg^−1^ Pb. This effect might be possibly due to the greater expression of transporter genes under such metal exposures, causing increased Fe absorption in the kidney and liver. Compared with Cu, Mn and Zn concentrations, the mice organ/tissue Fe concentration was more affected by the feed Pb and Cd spiking (Table S3). Similarly, Zhang et al. (2012) found that Cd injection increased the kidney Fe concentration, and the effect on Fe was greater than that on kidney Cu and Zn concentrations. Djukić-Ćosić et al. (2008) also found that mouse liver Fe concentration was increased by Cd injection. Similarly, Li et al. (2023b) found that exposure to low doses of Pb in drinking water resulted in elevated concentrations of Fe in the thighbone and teeth hippocampus of mice. Maintani and Suzuki (1986) found that liver Fe concentrations were more elevated than kidney Fe concentration under Cd exposure, similar to the current work.

The increase in mouse organ Fe concentrations with elevated Pb and Cd exposure may represent a mechanism by which mice mitigate the toxicity of these metals (Rybak et al. 2023). Higher Fe levels could enhance the competitive advantage of Fe over Cd or Pb for binding to active sites on proteins, thereby reducing the toxic effects of Cd or Pb. In this context, Fe homeostasis in mice may refer not only to the maintenance of absolute Fe levels but also to the stability of the ratio between Fe and competing elements. In this study, although organ Fe concentrations increased under Pb and Cd exposure (Table S3), the Fe/Cd ratio in organs decreased (Fig. S4), suggesting the occurrence of Fe deficiency. This may also explain why, despite the elevated organ Fe levels observed under Pb and Cd exposure, the expression of Fe-related transporter genes remained relatively high (Fig. 3 and Fig. 4). In order to confirm such a speculation, in the future, Fe deficiency symptoms should be monitored under such conditions.

### Transporter gene expression

Although synergism between cationic heavy metals have been observed in animals (Bulat et al. 2017; Ollson et al. 2017; Li et al. 2019, 2023a), the mechanism of such an effect is unknown. Ollson et al. (2017) speculated that the synergistic effect of Cd on the absorption of Pb in mouse liver may be due to the effect of the elevated concentration of Cd upregulating the expression of *Dmt1* in an effort to maintain Fe homeostasis, as *Dmt1* has a higher affinity for Cd than for Fe. The enhanced expression of *Dmt1* resulted in elevated synthesis of *Dmt1* which likely increased the transport of Pb, thus resulting in greater liver Pb concentrations under Cd exposure. However, this hypothesis has not been supported by experiments to date. In this work, positive effects of Cd on the liver *Dmt1* expression in the Cd and Pb co-exposed treatment (T14) as compared to the Pb-only treatment (T4) (Fig. 4A, Table  S4) and greater liver Pb concentration in the co-exposed treatment (T14) than the Pb-only treatment (T4) (Fig. 1B) were both observed. Thus, these results not only demonstrate the synergistic effect of Cd exposure on Pb accumulation in the mouse liver, but also support previous findings of this synergism as proposed by Ollson et al. (2017).

In the liver, the expression of *Zip14* was also upregulated by Cd exposure (Table S4). This may indicate that ZIP14 also has higher affinities for Cd than Fe, and both DMT1 and ZIP14 are involved in the transport and homeostasis of Fe in liver. It has been found that ZIP14 can transport Cd (Fujishiro et al. 2012; Jenkitkasemwong et al. 2012), and Fujishiro et al. (2017) found that ZIP14 had a higher affinity for Cd than DMT1. All these findings agree well with the results of this work. Furthermore, in the current work the expression of *Zip14* and *Dmt1* of T14 were 85.6 and 79% greater than those of T4, indicating a comparable effect of Cd exposure on these two genes.

The present work also found that the synergistic effect of Cd on Pb also existed in the kidney (between T14 and T4, Table  S4), and the expression of *Zip8* was upregulated by Cd exposure. Thus, the synergistic effect of Cd on Pb in the kidney might be due to the elevated expression of *Zip8* induced by Cd exposure. In agreement with these results, it has been found that ZIP8 exhibits a relatively high affinity for Cd (He et al. 2009; Gálvez-Peralta et al. 2012; Nebert et al. 2012), and the transport of Cd by ZIP8 has been confirmed in earlier studies (Fujishiro et al. 2009, 2012; Fujie et al. 2022). Moreover, Cd exposure is known to increase ZIP8 expression in mice (Schneider et al. 2014; Fujie et al. 2022). All these results agree well with the involvement of ZIP8 in the synergistic effect of Cd on Pb accumulation found in the present study.

However, explaining the relationship between the synergistic effect of Cd on Pb and the upregulation of *Zip8* and *Zip14* requires an additional premise, namely, that these transporters exhibit a higher affinity for Fe than for Cd. Indeed, the involvement of ZIP8 and ZIP14 in Fe transport in mammals has been confirmed (Jenkitkasemwong et al. 2012; van Raaij et al. 2019). For the affinity differences of these two transporters for different metals, Koike et al. (2017) found that affinities of different metals to ZIP8 ranked Fe = Zn > Cu = Cd = Co > Ni >  > Mg, Mn. Contrary to this, Nebert et al. (2012) found that the *V*_*max*_ of ZIP8 to different metals ranked Cd > Zn > Fe, in agreement with the results of the present work. For ZIP14, Girijashanker et al. (2008) found that transport of Cd by ZIP14 could not be inhibited by Fe, implying a higher affinity of ZIP14 for Cd than for Fe, in agreement with the results from the current work. Nebert et al. (2012) found that *V*_*max*_ of different metals for ZIP14A ranked Cd > Zn > Fe, and ZIP14B ranked Zn > Fe > Cd. These studies suggest the possibility that the similar Fe-induced compensation transporter gene expression for maintaining Fe homeostasis via DMT1 existed for ZIP8 and ZIP14 in the kidney and liver, respectively.

In a review article regarding ZIP8 and ZIP14 by Jenkitkasemwong et al. (2012), it was found that ZIP14 was abundantly expressed in liver, pancreas and heart, while ZIP8 was abundantly expressed in lung, testis and kidney. In the present work, under Cd exposure, upregulation of *Zip8* expression was found in the kidney, while the upregulation of *Zip14* expression was found in the liver (Table S4), with the latter in agreement with Jenkitkasemwong et al. (2012).

A synergistic effect of Pb on Cd was also observed in the kidney, liver and bone in this work (Table 1). Based on the speculation of Ollson et al. (2017), we think the synergistic effect of Pb on Cd may be due to Pb exposure inhibiting mouse absorption of an essential trace metal (possibly Fe) which shares the same transporter with Pb. Thus, in order to maintain homeostasis of this trace metal, expression of one or more genes were upregulated, and the transporters also transported Cd, thus resulting in elevated accumulation of Cd in mouse. However, in order to confirm such a speculation, one essential metal with lower affinities for these transporters, as compared to Pb, should exist. In this work, a synergistic effect of Pb on Cd were found between T14 and T11, and between T10 and T7 in the kidney (Table 1). However, no significant differences for the expression of *Dmt1*, *Zip8* and *Zip14* between T10 and T7, and between T14 and T11 were found (Fig. 3).

Literature suggests that DMT1 has a higher affinity for Fe than for Pb (Garrick et al. 2006; Słota et al. 2022). Thus, Pb exposure should have less effect on *Dmt1* expression than Cd exposure, meaning that Pb exposure should have a weaker effect on *Dmt1* expression than that of Cd exposure. This may be the reason why no significant upregulation of *Dmt1* expression was found for T10 as compared to T7, and for T14 as compared to T11 (Table S4, Fig. 3). Conversely, Bannon et al. (2002) found that DMT1 had similar affinities for Pb and Cd (with *Km* of 1.83 and 1.27 μmol L^−1^, respectively). Yet, Liu et al. (2020) found that Pb exposure reduced Fe accumulation in the midgut, similar to the liver Fe concentration affected by Pb doses in the current work (Table S3). These results imply that Pb exposure may have affected Fe homeostasis and the transporter gene expression, thus having a synergistic effect on mammal Cd accumulation. Such a synergism may have occurred in organ/tissues other than the kidney, such as in the intestines, as found in earlier works (Park et al. 2002; Breton et al. 2013; Ohta and Ohba 2020).

In the liver, the differences for *Dmt1* expression between T14 (108 mg kg^−1^ Pb + 1 mg kg^−1^ Cd) and T4 (108 mg kg^−1^ Pb), and between T4 and T1 (CK) suggest a positive effect of Cd and Pb exposure on *Dmt1* expression (Fig. 4). Meanwhile, greater liver Pb concentrations of T14 than T4, and of T4 than T1 were found (Fig. 1B); for Cd, greater liver concentrations were found for T14 than T4, while no significant difference was found between T4 and T1 (Fig. 2B). The liver Pb difference between T14 and T4 indicated that greater *Dmt1* expression helped with the accumulation of Pb in mouse liver, while the Cd concentrations of T4 and T1 indicated that the *Dmt1* expression did not help the transport of Cd in liver, thus there was a limited contribution of DMT1 on Cd transport in liver. Similarly, it has been confirmed that DMT1 plays an important role in Pb transport (Wang et al. 2011; Zhu et al. 2013; Villaseñor-Granados et al. 2019).

For the expression of *Zip14* in the liver, treatment T14 had significantly greater value than T4 (Fig. 4C), while treatment T14 had significantly greater liver Pb than T4 and T1 (Fig. 1B, Table S4). Thus, the greater liver Pb concentrations of treatment T14 as compared to T4 was likely due to the elevated expression of both *Dmt1* and *Zip14* in the liver under Cd exposure. This implies that the Cd exposure upregulated the expression of both genes in the liver. Previous evidence showed that DMT1 and ZIP14 are involved in Cd accumulation in animals, especially in intestine and kidney Cd absorption (Fujishiro et al. 2012, 2017), in agreement with the elevated *Dmt1* and *Zip14* expression in the liver under Cd exposure (Figs. 4A, C) in the current work. It is agreed that ZIP14 could transport multiple metals, including Zn, Fe, Cd and Mn (Aydemir and Cousins 2018); results of the present work further indicate that ZIP14 may also transport Pb in mouse. Both DMT1 and ZIP14 exist in liver and play an important role in Fe transport (Nam et al. 2013). It has been found that ZIP14 is ubiquitous but often higher in liver than in kidney (Schneider et al. 2014; Aydemir and Cousins 2018). This may be the reason why the Cd and Pb exposure resulted in a ZIP14 expression increase in the liver (Fig. 4C) but not in the kidney (Fig. 3C).

The decreased Zn and Mn concentrations in the kidney and liver of the treatments with greater Cd or/and Pb doses than the CK (Table S3) may be due to the competition between the spiked Pb and Cd in feed and Zn and Mn for transporters in the kidney and liver, as found in previous works (Bulat et al. 2008; Li et al. 2019, 2023a). Furthermore, the elevated kidney and liver Fe concentrations of some of the Cd/Pb spiked treatments as compared to the CK agreed well with the transporter gene expression regulation (Fig. 3and Fig. 4) and the mouse organ Pb and Cd concentration (Fig. 1 and Fig. 2), suggesting a possible synergistic effect on Fe in liver and kidney by feed Cd and Pb spiking. The significant positive correlation between Cd and Fe concentrations in the kidney (Fig. S3A) may be due to the elevated expression of *Zip8* under Cd exposure (Fig. 3B) helped the accumulation of Fe in the kidney; the elevated expression of *Dmt1* (Fig. 4A) and *Zip14* (Fig. 4C) in liver under Cd exposure may have led to Fe accumulation in the liver, resulting in a significant correlation between liver Cd and Fe concentrations (Fig. 3B). Such differences of the effects of the feed Cd and Pb spiking on mouse Fe, Zn and Mn concentrations suggest that Fe may have a higher affinity for DMT1 and ZIP14 than Zn and Mn. In agreement with this, Garrick et al. (2006) suggested that the affinity of different metals to DMT1 were Mn > ?Cd > ?Fe > Pb-Co–Ni > Zn, while Mackenzie et al. (2007) also agreed that DMT1 had stronger affinities for Fe than Zn.

The accumulation of trace metals in mammal organs such as the liver and kidney is governed by the multiple processes including intestinal absorption (particularly in the duodenum and jejunum), organ-specific uptake from the circulation, and subsequent excretion (Scindia et al. 2019; Chen et al. 2024). Therefore, future studies on Cd and Pb interactions in mammals should also take into account the expression of transporter-related genes in both target organs and the intestine, as well as genes responsible for metal efflux from these organs.

## Conclusions

Analysis of Pb and Cd concentrations in mice indicated that a synergistic effect of Pb on Cd occurred in all three organ/tissues analyzed. Synergistic effects of Pb on Cd were found in the kidney and bone, while synergistic effects of Cd on Pb were found in the kidney and liver. The organ/tissues Pb and Cd concentrations between the synergistic pairs of treatments increased by between 10–230%, confirming the synergisms between these two metals. Transporter gene expression analysis of the synergistic treatments indicated that *Zip8* expression in the kidney was upregulated by Cd exposure, while *Dmt1* and *Zip14* expression in the liver was upregulated by the Cd and Pb exposure and Cd co-exposure, respectively. The upregulated *Zip8* in the kidney and *Dmt1* and *Zip14* in the liver explained the synergized Pb in both organs. The synergized Cd in the kidney may be due to the upregulation of other transporter genes or to the upregulation of transporter genes in non-studied organs such as the intestines. Overall, this study advances our understanding of the rarely observed synergistic interactions among cationic heavy metals and sheds new light on their underlying mechanisms of metal accumulation in mice and possibly other animals.

## Supplementary Information

Below is the link to the electronic supplementary material.

Supplementary file1 (DOCX 1157 KB)

## Data Availability

All the data have been deposited with Science Data Bank (10.57760/sciencedb.34805).
