# Supplementary material for: Synergistic interaction between lead and cadmium via metal-transporter gene upregulation in mice
Source: Biometals. 2026 May 17;39(5):1767–81. doi: 10.1007/s10534-026-00829-3 (PMC13518445; doi:10.1007/s10534-026-00829-3)
Supplement: Supplementary file 1 [file 10534_2026_829_MOESM1_ESM.docx]

Synergistic interaction between lead and cadmium exposure via metal-transporter gene upregulation in mice

Liping Li ^a, c^, Tingting Zhang ^a, c^, James A. Ippolito ^b^, Shuangshuang Shao ^d^, Weiqin Xing ^a, c,*^, Xinjun Huang ^a, c^, Yongqiang Yang ^a, c^, Linlin Zhao ^e^, Yongxia Cheng ^e^

^a^ School of the Environment, Henan University of Technology, Zhengzhou, Henan 450001, China

^b^ School of Environment and Natural Resources, The Ohio State University, Columbus, OH 43210, USA

^c^ Henan International Joint Laboratory of Environmental Pollution, Remediation and Food Quality Security, Zhengzhou, Henan 450001, China

^d^ School of Environmental and Bioengineering, Henan University of Engineering, Zhengzhou, Henan 451191, China

^e^ Jiyuan Ecological and Environmental Monitoring Center of Henan Province, Jiyuan, Henan 459000, China

*Corresponding authors, Weiqin Xing, xingwq@haut.edu.cn

ORCID

Tingting Zhang, 0009-0000-7121-8910; Liping Li: 0000-0003-0589-5336; James A. Ippolito: 0000-0001-8077-0088; Shuangshuang Shao, 0000-0001-8581-1010; Weiqin Xing: 0000-0001-7557-9940; Yongqiang Yang: 0000-0002-7109-6164; Xinjun Huang: 0000-0002-6851-4716

**Fig. S1** Correlation of heavy metal concentrations in the Kidney (A), liver (B) and bone (C) of mice fed diets spiked with different levels of lead and cadmium (treatment T1-T16, *n*=4). * indicates significance at a *p* < 0.05

**Fig. S2** Correlation between Fe and Cd molar ratios and Cd concentrations in the kidney (A), liver (B) and bone (C) of mice fed diets spiked with different levels of lead and cadmium (*n*=64)

**Fig. S3** Dose response of Pb accumulation concentrations in mouse organ/tissues after daily diet of 15-108 mg kg^-1^ (2410-1769 µg kg^-1^ body weight d^-1^) Pb (Ac)_2_ and 1-3 mg kg^-1^ (250-5760 μg kg^-1^ body weight d^-1^) Cd(Cl)_2_ for 10 days (*n*=12). (■: Cd=0, Pb=15, 30 and 108 mg kg^-1^; ●: Cd=1, Pb=15, 30 and 108 mg kg^-1^; ▲: Cd=2, Pb=15, 30 and 108 mg kg^-1^; ▼: Cd=3, Pb=15, 30 and 108 mg kg^-1^)

**Fig. S4** Dose response of Cd accumulation concentrations in mouse organ/tissues after daily diet of 15-108 mg kg^-1^ (2410-1769 µg kg^-1^ body weight d^-1^) Pb (Ac)_2_ and 1-3 mg kg^-1^ (250-5760 μg kg^-1^ body weight d^-1^) Cd(Cl)_2_ for 10 days (*n*=12). (■: Cd=0, Pb=15, 30 and 108 mg kg^-1^; ●: Cd=1, Pb=15, 30 and 108 mg kg^-1^; ▲: Cd=2, Pb=15, 30 and 108 mg kg^-1^; ▼: Cd=3, Pb=15, 30 and 108 mg kg^-1^)

Table S1. Descriptive statistics of total Cd and Pb concentrations in feed of different treatments. Data are presented as mean ± SE.

| Treatment | Feed, metal added and dose (mg kg^-1^) | Diet concentration | |  | Mouse metal intake | | | |
| --- | --- | --- | --- | --- | --- | --- | --- | --- |
|  |  | Cd | Pb |  | Cd | | Pb | |
|  |  | (mg kg^-1^ DW) | |  | (mg kg^-1^ BW day^-1^) | | | |
| T1 | Basal Feed (BF) | 0.240 ± 0.01 | 0.591 ± 0.060 |  | |  | |  |
| T2 | BF, Pb 15 | 0.296 ± 0.010 | 22.3 ± 0.9 |  | | 2.41 | |  |
| T3 | BF, Pb 30 | 0.205 ± 0.001 | 34.1 ± 1.2 |  | | 5.11 | |  |
| T4 | BF, Pb 108 | 0.294 ± 0.01 | 111 ± 1.65 |  | | 17.7 | |  |
| T5 | BF, Cd 1 | 1.54 ± 0.08 | 1.09 ± 0.36 | 0.250 | |  | |  |
| T6 | BF, Cd 2 | 2.55 ± 0.06 | 0.667 ± 0.030 | 0.441 | |  | |  |
| T7 | BF, Cd 3 | 3.80 ± 0.06 | 1.46±0.03 | 0.576 | |  | |  |
| T8 | BF, Pb 15, Cd 1 | 1.52 ± 0.04 | 17.7±1.07 | 0.242 | | 2.80 | |  |
| T9 | BF, Pb 15, Cd 2 | 2.52 ± 0.06 | 18.1±0.02 | 0.412 | | 2.96 | |  |
| T10 | BF, Pb 15, Cd 3 | 3.84 ± 0.10 | 19.3±0.39 | 0.633 | | 3.18 | |  |
| T11 | BF, Pb 30, Cd 1 | 1.44 ± 0.05 | 36.9 ± 0.51 | 0.239 | | 6.09 | |  |
| T12 | BF, Pb 30, Cd 2 | 2.62 ± 0.03 | 36.7 ± 0.65 | 0.416 | | 5.82 | |  |
| T13 | BF, Pb 30, Cd 3 | 3.85 ± 0.09 | 34.8 ± 1.28 | 0.650 | | 5.87 | |  |
| T14 | BF, Pb 108, Cd 1 | 1.81±0.05 | 137 ± 1.84 | 0.308 | | 23.4 | |  |
| T15 | BF, Pb 108, Cd 2 | 2.70 ± 0.10 | 124 ± 1.36 | 0.460 | | 21.1 | |  |
| T16 | BF, Pb 108, Cd 3 | 3.86 ± 0.09 | 124 ± 1.16 | 0.648 | | 20.7 | |  |

Table S2. Primer sequences and amplicon information.

| Gene |  | Primers (5´~3´) | Length | Tm |
| --- | --- | --- | --- | --- |
| *Dmt1* | Forward | CAATGTCTTTGTCGTGTCCGT | 21 | 60.8 |
|  | Reverse | GCGACCATTTTAGGTTCAGGAAT | 23 | 60.9 |
| *Zip8* | Forward | GCCAAGCTCATGTACCTGTCT | 21 | 61.8 |
|  | Reverse | AAGATGCCCCAATCGCCAA | 19 | 62.0 |
| *Zip14* | Forward | GTGTCTCACTGATTAACCTGGC | 22 | 60.6 |
|  | Reverse | AGAGCAGCGTTCCAATGGAC | 20 | 62.5 |

Table S3. Mean c**oncentrations of Cu, Zn, Mn and Fe in mouse organs and tissues of each treatment.** Different letters of the same metal in the same organ/tissue indicate significant difference at *p* *<* 0.05.

| Feed and spiked metal (mg kg^-1^) | | Metal concentration (mg kg^−1^) | | | | | | | | | | | | | |
| --- | --- | --- | --- | --- | --- | --- | --- | --- | --- | --- | --- | --- | --- | --- | --- |
|  |  | Kidney | | | |  |  | Liver | | |  |  | Bone | |  |
|  |  | Cu | Zn | Mn | Fe |  | Cu | Zn | Mn | Fe |  | Cu | Zn | Mn | Fe |
| T1 | Basal Feed (BF) | 11.4bc | 102a | 5.70ab | 277cde |  | 19.4a | 201a | 8.18abc | 474fg |  | 1.31a | 253a | 3.54ab | 251ab |
| T2 | BF, Pb 15 | 11.2bc | 103a | 5.36abc | 313abcde |  | 19.3a | 182ab | 7.93abc | 434g |  | 1.02bcd | 256a | 2.63bc | 246ab |
| T3 | BF, Pb 30 | 11.4bc | 95.5abc | 5.32abc | 302bcde |  | 19.6a | 189ab | 8.79a | 510defg |  | 0.785d | 263a | 2.29cde | 227ab |
| T4 | BF, Pb 108 | 12.1ab | 101ab | 5.16abcd | 289cde |  | 19.6a | 189ab | 7.89abc | 496defg |  | 0.881bcd | 239a | 2.71bc | 230ab |
| T5 | BF, Cd 1 | 10.7c | 97.7abc | 5.03abcd | 389ab |  | 19.3a | 194ab | 7.74abc | 489efg |  | 1.0bcd | 253a | 2.47cd | 232ab |
| T6 | BF, Cd 2 | 10.9b | 95.0abc | 5.08abcd | 340abc |  | 19.0a | 201a | 8.47abc | 566bcdef |  | 1.16ab | 250a | 3.09a | 273a |
| T7 | BF, Cd 3 | 11.0bc | 94.4abc | 5.44abc | 400a |  | 18.8a | 197ab | 8.56ab | 677a |  | 0.924bcd | 251a | 3.51ab | 267ab |
| T8 | BF, Pb 15, Cd 1 | 10.7c | 89.2bcd | 5.33abc | 260cde |  | 18.8a | 190ab | 7.96abc | 602abcd |  | 1.03abcd | 247a | 2.52cd | 216ab |
| T9 | BF, Pb 15, Cd 2 | 10.8bc | 80.9de | 5.08abcd | 241de |  | 16.5a | 183ab | 7.42bc | 516defg |  | 0.907bcd | 257a | 2.10cde | 202ab |
| T10 | BF, Pb 15, Cd 3 | 10.8bc | 75.8e | 5.27abc | 254cde |  | 16.4a | 182ab | 7.18c | 535cdefg |  | 1.10abc | 268a | 2.08cde | 231ab |
| T11 | BF, Pb 30, Cd 1 | 10.9bc | 80.6de | 4.97bcd | 219e |  | 17.5a | 187ab | 7.16c | 505defg |  | 0.889bcd | 232a | 1.98cde | 203ab |
| T12 | BF, Pb 30, Cd 2 | 11.6bc | 86.4cde | 5.71ab | 297bcde |  | 18.4a | 199ab | 8.33abc | 532cdefg |  | 1.13ab | 251a | 2.09cde | 236ab |
| T13 | BF, Pb 30, Cd 3 | 11.5bc | 89.0bcd | 4.62cd | 300bcde |  | 16.4a | 172b | 7.26bc | 621abc |  | 0.827cd | 262a | 1.86cde | 236ab |
| T14 | BF, Pb 108, Cd 1 | 11.4bc | 87.0cd | 4.29d | 309bcde |  | 15.8a | 175ab | 7.46abc | 591abcde |  | 0.941bcd | 247a | 1.56de | 238ab |
| T15 | BF, Pb 108, Cd 2 | 12.9a | 95.7abc | 5.45abc | 318abcd |  | 16.4a | 178ab | 7.46abc | 638ab |  | 1.17ab | 237a | 1.47e | 198b |
| T16 | BF, Pb 108, Cd 3 | 13.2a | 103a | 5.99a | 292cde |  | 16.5a | 180ab | 7.20bc | 648ab |  | 0.991bcd | 232a | 1.54de | 209ab |

Table S4. Transporter gene upregulation and the potential inducing metals.

| Organ | Group | Treatment | Metal spiked (mg kg^-1^) | Synergized metal, %increase | Gene upregulation, inducing metal | | |
| --- | --- | --- | --- | --- | --- | --- | --- |
|  |  |  |  |  | *Dmt1* | *Zip8* | *Zip14* |
| Kidney | I | T4 | Pb 108, Cd 0 | Pb, 10.6% | No | Yes, Cd | No |
|  |  | T14 | Pb 108, Cd 1 |  |  |  |  |
|  | II | T7 | Pb 0, Cd 3 | Cd, 49.2% | No | No | No |
|  |  | T10 | Pb 15, Cd 3 |  |  |  |  |
|  | III | T11 | Pb 30, Cd 1 | Cd, 132% | No | No | No |
|  |  | T14 | Pb 108, Cd 1 |  |  |  |  |
| Liver | IV | T4 | Pb 108, Cd 0 | Pb, 60.2% | Yes, Cd | No | Yes, Cd |
|  |  | T14 | Pb 108, Cd 1 |  |  |  |  |
